# *Fissistigma oldhamii* (Hemsl.) Merr.: Ethnomedicinal, Phytochemistry, and Pharmacological Aspects

**DOI:** 10.3390/plants12244094

**Published:** 2023-12-07

**Authors:** Christian Bailly

**Affiliations:** 1 CNRS, Inserm, CHU Lille, UMR9020-U1277-CANTHER—Cancer Heterogeneity Plasticity and Resistance to Therapies, OncoLille Institut, University of Lille, 59000 Lille, France; christian.bailly@univ-lille.fr; 2 Institute of Pharmaceutical Chemistry Albert Lespagnol (ICPAL), Faculty of Pharmacy, University of Lille, 59006 Lille, France; 3 OncoWitan, Scientific Consulting Office, 59290 Lille, France

**Keywords:** anticancer, anti-inflammatory, *Fissistigma oldhamii*, phytochemicals, traditional medicine

## Abstract

The species *Fissistigma oldhamii* (Hemsl.) Merr. (Annonaceae) has long been used as a traditional herbal medicine in China to treat diverse human diseases. Decoctions from the roots of the plant (Guā Fù Mù) are used to treat body pain and inflammatory pathologies, such as rheumatic syndromes, sciatica, and osteoarthritis. The phytochemical content of the plant and the associated pharmacological activities have been analyzed. Seventy natural products were identified in the different parts of the plants, namely, the roots, stems, leaves, fruits, and seeds. The compounds comprise many tri- and tetracyclic alkaloids (aporphine-type), anthraquinones, terpenoids, flavonoids, and others. The pharmacological properties of these molecules were analyzed to point out the anti-inflammatory, antioxidant, anticancer, and/or antimicrobial effects, together with the underlying modulated pathways and molecular targets in some cases. The panel of phytoconstituents present in *F. oldhamii* extracts is large, with the majority of bioactive products identified in the roots and stems. Multiple molecules can contribute to the anti-inflammatory properties of the extracts. Network pharmacology analyses of the phytoconstituents are needed to better delineate the effective components and their targets.

## 1. Introduction

The Annonaceae is the largest family in the order Magnoliales with about 120 genera, including the genus *Annona*, which contains about the same number (120) of species of trees, bushes, climbers, and shrubs with a worldwide distribution [1]. Many Annona species have been largely studied due to their medicinal properties and their specific contents of bioactive secondary metabolites [2]. This is the case for the genus *Fissistigma*, which includes diverse plants used in traditional medicine, such as *Fissistigma glaucescens*, *F. polyanthoides*, *F. retusum*, and others, mainly distributed in tropical areas of Asia [3,4]. Among these plants, the species *F. oldhamii* has received less attention thus far.

**Figure 1:**
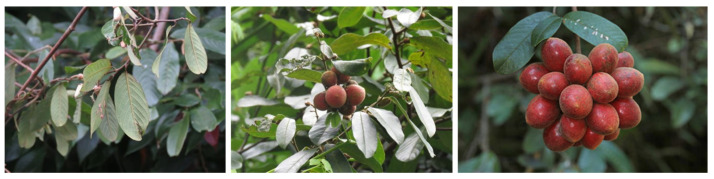
The plant *Fissistigma oldhamii* (Hemsl.) Merr. with green leaves, stems, and ripe red fruits (https://www.gbif.org/fr/occurrence/gallery?taxon_key=3157344) accessed on 6 September 2023.

*Fissistigma oldhamii* (Hemsl.) Merr. (synonyms: *Melodorum oldhamii* Hemsl., *Fissistigma oldhamii* var. *longistipitatum*) is a climbing shrub with simple broad and thick leaves (Figure 1). The plant is well distributed in south-east China (Fujian, Guangdong, Guangxi, Hainan, Hunan, Jiangxi, Taiwan, SE Yunnan, S Zhejiang provinces) and North Vietnam. It is a plant listed in China as threatened with extinction or endangered because of the increasing forest exploitation and destruction [5]. The plant has a characteristic pollen morphology with coarsely regulated ornamentation [6]. This species is the preferred host plant for the ephemeral butterfly *Graphium agetes* (a Lepidopteran also known as the four-bar swallowtail butterfly), which uses the folded immature leaves to lay and embed one or two creamy eggs [7].

*Fissistigma oldhamii* liana grows primarily in the subtropical biome. It is a middle-sized vine also known for its flowers, yielding a perfumed oil used in cosmetics, and for its fruits ripening to red and being edible and tasty [8]. The plant oil is used as a lubricant and for manufacturing soap. The fibers obtained from the inner bark can be used to make rope, sacks, and paper. *F. oldhamii* is a medicinal plant traditionally used to treat diverse human diseases. The roots of the plant, known as “Guā Fù Mù” in Chinese (radix *Fissistigmatis oldhamii*), are used as a decoction, infusion, or bath to treat rheumatism syndromes and body pain, to promote blood circulation, or to treat skin infections. The product has the effect of dispelling wind to eliminate dampness, according to the principles of the Chinese pharmacopeia. The leaves and stems can be used to treat gynecological inflammation and rheumatism [9]. The medicinal properties can be attributed to the presence of diverse bioactive natural products in plant decoctions and extracts used therapeutically.

In the subsequent section, we shall review the diversity of natural products found in the most commonly used plant parts and their pharmacological properties. The objective of the analysis is twofold. The first objective is to offer an updated view of the bioactive products identified thus far from *F. oldhamii* and their pharmacological properties. The second goal is to provide the molecular basis of the anti-rheumatic action of the plant. A thorough literature search was performed up to October 2023 using multiple databases and different keywords. However, it is a non-exhaustive exploration essentially targeting publications, reports, and patents, mainly in the English language.

## 2. Phytochemical Content of *Fissistigma oldhamii*

Natural products have been isolated from different parts of the plant. There are studies using the roots—radix *Fissistigmatis oldhamii*—which are the essential interface between the plant and the soil for the capture of resources. Many products derive from the foliage and stems of the plant, while others come from the fruits and seeds. Each plant part has been analyzed independently (Table 1).

### 2.1. Natural Products from the Roots

**Figure 2:**
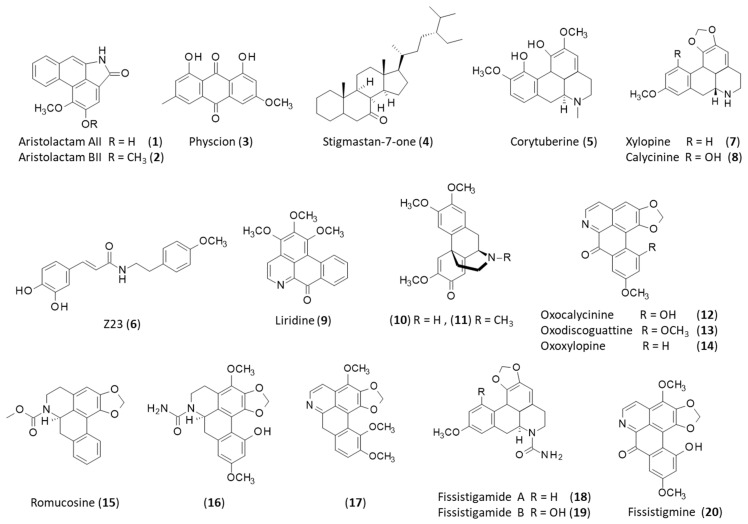
Selected natural products isolated from *F. oldhamii.* Structures of compounds **1**–**20**. See Table 1 for details.

The roots and rhizomes of *F. oldhamii* (Haifengteng) have been used in TCM essentially to cure arthritis, chest pain, stomach pain, and liver injuries [10]. The alkaloids aristolactams A and B (**1**–**2**) were among the first compounds characterized from the roots of *F. oldhamii* about thirty years ago (Figure 2). Aristolactam B (now designated aristolactam BII (**2**), also known as cepharanone B) displays antiproliferative activity, whereas aristolactam A (aristolactam AII (**1**)) is essentially an inactive product. For example, aristolactam B markedly inhibits the proliferation of HCT-15 colonic epithelial cells, whereas aristolactam A revealed no activity (IC_50_ = 5.5 and >40 µM, respectively) [28]. Aristolactam B displays pronounced cell-line selectivity. It is a potent antiproliferative agent against XF 498 human CNS cells (IC_50_ = 0.84 µM), moderately active against SK-OV-3 ovarian cancer cells (IC_50_ = 8.3 µM), and poorly effective against A549 lung cancer cells (IC_50_ = 23.2 µM) [28]. This compound has been used as a template for the design and synthesis of analogs endowed with potent antitumor activities against a broad array of cancer cell lines [29]. Other lactams have been identified in the plant roots. A Chinese patent (CN102477039A) describes the procedure to obtain lactam compounds from the dried powdered roots of the plant upon extraction with ethanol and then dichloromethane. Another recent Chinese patent discloses a preparation method for a root extract using supercritical carbon dioxide (CN113350827A).

Aristolactams A-B were co-isolated from the plant roots together with the anthraquinone derivative physcion (**3**) and the sterol derivative stigmastan-7-one (**4**) [11]. Physcion (Figure 2), a natural product found in many plants (for example, in rhubarb), is known for its capacity to reduce oxidative stress and endoplasmic reticulum stress through the activation of the eNOS/Nrf2 signaling pathway and the inhibition of the JAK2/STAT3 pathway [30,31]. It could be a useful product to protect against cerebral ischemia–reperfusion injury [32]. This compound has also shown modest antiproliferative activity against cervical and breast cancer cells via the modulation of oxidative-stress-mediated mitochondrial apoptosis [33,34]. It exhibits marked antibacterial activity, notably against the veterinary pathogen Chlamydia psittaci, which is responsible for psittacosis (or parrot fever) in humans [35]. It is also a useful compound able to promote hair growth through the inhibition of 5α-reductase (IC_50_ = 191.9 μM). It is therefore of potential interest for treating androgenic alopecia [36].

Other natural products have been identified in the plant roots, such as the aporphine-type alkaloid corytuberine (**5**) [10]. This compound is an antibacterial agent targeting malonyl-CoA:acyl carrier protein transacylase (MCAT) from *Helicobacter pylori*, which can cause chronic gastric inflammation and gastric cancer [37,38].

The immunosuppressive compound coded Z23 (**6**) (N-caffeoyl O-methyltyramine) has been isolated from *F. oldhamii* using both the roots and stems of the plant. This propenamide derivative was shown to block concanavaline A-induced proliferation of splenocytes (IC_50_ = 6.22 µM) while being non-cytotoxic even at a high concentration of 100 µM. It inhibited anti-CD3/28 mAb-induced T-cell proliferation in a dose-dependent manner, thereby reducing the production of the cytokines interleukine-2 (IL-2) and interferon-γ (IFN-γ) released by T-cells. Its suppressive effect on the T-cell-dependent immune response was evidenced in vivo using a model of collagen-induced arthritis (CIA) in mice. At a dose of 25 mg/kg (ip, once daily for 2 weeks), the compound markedly reduced the incidence and severity of CIA, and its effect was associated with a profound reduction in the production of IL-2 and IFN-γ. This compound is a potent suppressor of T-cell activation and an inhibitor of Th1-type cytokine production [12]. A subsequent study revealed that Z23 (**6**) reduced the production of several inflammatory mediators and cytokines in lipopolysaccharide (LPS)-stimulated RAW264.7 macrophages, notably nitric oxide (NO), prostaglandin E2 (PGE2), tumor necrosis factor-α (TNF-α), and IL-6. It also diminished the expression of inducible nitric oxide synthase (*iNOS*) and cyclooxygenase-2 (*COX2*) genes, thus confirming its potential value for the treatment of inflammatory diseases such as rheumatoid arthritis [39]. Exactly the same compound has been isolated from the plant *Cuscuta reflexa* Roxb. (Convolvulaceae), a climber commonly found in Pakistan and used medicinally for the treatment of liver damage. In this case, the isolated compound was named *Cuscuta* propenamide 1, but it is the same molecule with a catechol-enamide scaffold. This compound was isolated together with related analogs and was shown to modestly inhibit α-glucosidase (IC_50_ = 103.6 µM) [40]. Mono- and bis-glucosylated derivatives of this product can be found in nature. *C. reflexa* is a well-known hepatoprotective plant containing a variety of bioactive natural products [41]. Z23 (**6**) is one of the key anti-inflammatory products from *C. reflexa* [42].

### 2.2. Natural Products from the Stems

Most natural products isolated from *F. oldhamii* come from the stem parts. Earlier work reported the isolation of five alkaloids: the two aporphine alkaloids xylopine (**7**) and calycinine (**8**), the oxoaporphine alkaloid O-methylmoschatoline (**9**) (also known as homomoschatoline or liridine (**9**)), and the two morphinandienone alkaloids N-methyl-2,3,6-trimethoxymorphinandien-7-one (**10**) and N-nor-2,3,6-trimethoxymorphinandien-7-one (**11**) [43], which are analogs of salutaridine (see below). Morphinandienone alkaloids often display marked anti-inflammatory properties [44]. At that time, only the structural characterization of the natural products was reported, but they were characterized later from a pharmacological viewpoint. Like other aporphine alkaloids, xylopine (**7**) displays anticancer properties via the induction of oxidative stress and G2/M cell cycle arrest in cancer cells [45]. Calycinine (**8**) (also known as fissistigine A or fissoldine) is a rarer compound and much less cytotoxic than xylopine [46,47]. A derivative designated oxocalycinine (**12**) has been isolated together with the close analog oxodiscoguattine (**13**), with the former being more potent than the latter in inhibiting both B- and T-cell proliferation, but it is also a more cytotoxic compound [48]. The plant offers a rich content of (oxo)aporphine alkaloids of all types, such as the products xylopine (**7**) and oxoxylopine (**14**) (also known as lanuginosine), as well as the known products O-methylmoschatoline (**9**) and romucosine (**15**) [49]. The latter N-methoxycarbonyl aporphine alkaloid exerts inhibitory effects on platelet aggregation induced by platelet-activating factor (PAF) or arachidonic acid [50]. It has also revealed antifungal activities against several phytopathogenic fungi, such as the species Alternaria kikuchiana Takana (EC_50_ = 0.316 g/L), which produces black spot disease in pears [51].

**Figure 3:**
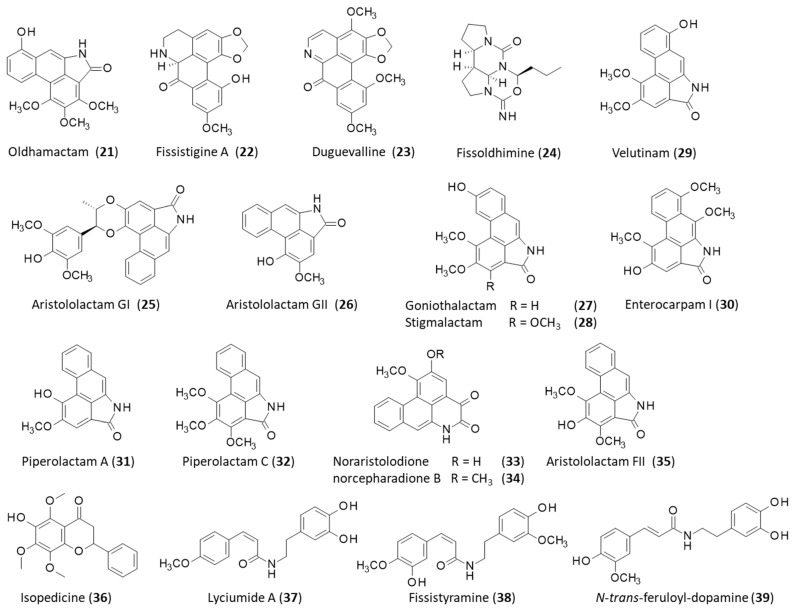
Structures of compounds **21**–**39**.

Novel aporphine alkaloids are regularly identified in *F. oldhamii*. For example, a recent study reported the isolation and antiproliferative action of two new products from the dried stems, namely, (R)-1,2-methylenedioxy-3,9-dimethoxy-11-hydroxy-N-carbamoyl-noraporphine (**16**) and 3,10,11-trimethoxy-1,2-methylenedioxy-7-oxoaporphine (**17**), isolated together with the known related products fissistigamides A (**18**) and B (**19**), fissistigmine (**20**), and oldhamactam (**21**) (Figure 3) [13]. It is worth noting that oldhamactam is one of the many compounds predicted to bind to the human enzyme IKK-2 (inhibitor NF-kB kinase 2), which participates in the process of NF-κB activation in response to various inflammatory stimuli [52]. The plant contains other uncommon oxoaporphine alkaloids, such as fissistigine A (**22**) and duguevalline (**23**), which have been rarely described in other species [53,54]. The latter compound is an aporphinoid originally found in the stems of Duguetia vallicola J.F.Macbr. and also found in Dasymaschalon blumei Finet & Gagnep. [55]. It has revealed modest antiproliferative activity against P388 leukemia cells (IC_50_ = 9.4 µM) [56]. Fissistigine A (**22**) (fissoldine or (−)-calycinine [46]) has been shown to inhibit collagen-induced platelet aggregation [57,58].

The aforementioned product O-methylmoschatoline (liridine (**9**)) has been found in a few plants, notably in the leaves of Xylopia sericea and the branches of Annona foetida. It has shown little antiproliferative activity and was found to be inactive against the malaria parasite [59,60]. It has revealed mild activity against the trypomastigote forms of Trypanosoma cruzi, the pathogen responsible for Chagas disease (EC_50_ = 3.8 μg/mL) [59]. The compound also displays modest antibacterial properties against both Gram-positive and -negative strains, together with weak antifungal action [61]. In particular, this oxoaporphine alkaloid (**9**) was found to be active against Staphylococcus epidermidis (strain 6 ep, MIC = 25.0 µg/mL) and against Candida dubliniensis (strains ATCC 777 and ATCC 778157, MIC = 12.5 and 25.0 µg/mL, respectively) [62].

The alkaloid fissistigmine A (**20**) has been found in the stem of *F. oldhamii* [14] and the related species Fissistigma tungfangense Y.Tsiang & P.T.Li. This compound inhibited the proliferation of synoviocytes in vitro with a potency comparable to the reference product methotrexate (IC_50_ = 114.6 and 112.8 µM, respectively), suggesting its potential use for the treatment of rheumatoid arthritis [63]. Fissistigmine (**20**) has been isolated from the stem part of the plant together with the related aporphine alkaloids fissistigamides A-B (**18**–**19**) [14]. These two compounds have never been described in other plants. The unrelated tetracyclic alkaloid fissoldhimine (**24**), isolated from the fresh stems of *F. oldhamii*, is possibly biosynthetically derived from the multistep oxidation of putrescine [15]. The total synthesis of this compound based on the heterodimerization of pyrroline has been described, but there are no biological data associated with this atypical alkaloid (**24**) (Figure 3) [64].

Two other important products isolated from the stems of *F. oldhamii* are aristololactams GI (**25**) and GII (**26**), which are both anti-inflammatory products able to reduce the production of cytokines IL-6 and TNF-α in lipopolysaccharide-stimulated RAW264 murine macrophages. Their effect was not spectacular (20–30% inhibition at 10 µM, compared to 80% inhibition with the control parthenolide) but was significant, with aristololactam GII (**26**) being more active than aristololactam GI (**25**) [14]. Aristololactam GI is an atypical aporphinoid–lignan hybrid compound bearing an aristolactam scaffold linked to a phenylpropanoid unit via a benzodioxane ring. Its stereoselective total synthesis has been reported [65]. Other aristololactam-type alkaloids have been identified, including several aristololactams (AII, AIIIa, BII, FI, FII) and the analogs goniothalactam (**27**), stigmalactam (**28**), velutinam (**29**), and enterocarpam I (**30**), together with piperolactams A (**31**) and C (**32**) and two dioxoaporphines, noraristolodione (**33**) and norcepharadione B (**34**) [14,66]. The latter compound (also found in the medicinal plant *Houttuynia cordata* Thunb. (HC)) has been shown to protect cells from oxidative stress induced by hydrogen peroxide via a dual mechanism: the upregulation of heme oxygenase 1 (HO-1, dependent on PI3K/Akt signaling) and a reduction in the activation of volume-sensitive outwardly rectifying (VSOR) Cl^−^ channels. It is viewed as a useful compound in protecting neurons or repairing neuronal injury in the context of neurodegenerative diseases or stroke [67].

Aristololactams are present in many plant species, notably those in the Aristolochiaceae family. They constitute a large group of products with anticancer and anti-inflammatory properties, but they are also associated with occasional nephrotoxic and carcinogenic effects [68,69]. Aristolochic acid nephropathy (ANN) is a chronic kidney disease associated with carcinoma of the upper urinary tract, mostly prevalent in China and other Asian countries due to the extended use of Aristolochia herbs [70]. Interestingly, aristolactam FII (**35**) has revealed significant inhibitory effects against platelet aggregation induced by arachidonic acid, collagen, or platelet-activating factor (PAF), possibly via the inhibition of the formation of thromboxane A2 [16].

Flavonoids have also been characterized from the stem of *F. oldhamii*, such as the flavanone derivative isopedicin (**36**), characterized as a potent inhibitor of the production of superoxide anions (*O*_2_^•−^) in activated human neutrophils (IC_50_ = 0.34 µM) [17]. This compound can also be found in Didymocarpus pedicellata R. Br. (Gesneriaceae), an antioxidant plant widely used in traditional Indian medicine [23,71,72]. Isopedicin (**36**) is not a direct inhibitor of NADPH oxidase but an inhibitor of the formation of *O*_2_^•−^ via the adenosine/cAMP pathway and the specific inhibition of phosphodiesterase [17]. This type of compound could be useful in treating diverse neutrophil-associated inflammation diseases, such as hemorrhagic-shock-induced lung injury, for example. Flavonoids can be found in all parts of the plant. A procedure has been refined to optimize their extraction from the whole plant [73].

**Figure 4:**
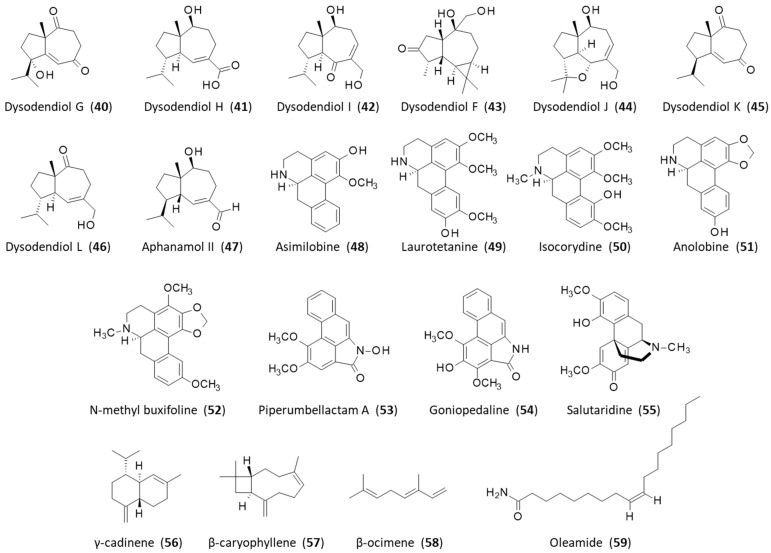
Structures of compounds **40**–**59**.

Five coumaroyltyramine derivatives have been characterized recently from the stem of the plant, including the two analogous products lyciumide A (**37**) and fissistyramine (**38**) [18]. The former is a dopamine derivative, originally discovered in the fruits of the medicinal plant Lycium barbarum L. (goji berry) [74], and can be found in the stems of Lycium arabicum Schweinf. ex Boiss. [75]. It is an antioxidant compound. The latter molecule, fissistyramine, has been found only in *F. oldhamii* and displays a modest capacity to inhibit the proliferation of synoviocytes comparable to that of lyciumide A (IC_50_ = 12.1 and 13.8 µM, respectively). A similar level of activity was evidenced with the related product N-trans-feruloyl-dopamine (**39**) (IC_50_ = 15.6 µM) [18]. In fact, the plant stems contain a variety of natural products contributing to the inhibition of synoviocyte proliferation, including a weakly active fatty acid methyl ester (IC_50_ = 38.6 µM) [76] and guaiane-type sesquiterpenoids, such as dysodensiols G-I (**40**–**42**), isolated for the first time from *F. oldhamii*. Dysodensiol I (**42**) was shown to potently inhibit synoviocyte proliferation (IC_50_ = 1.0 µM), whereas dysodensiols G and H were considerably less active in the same assay (IC_50_ = 10.2 and 52.4 µM, respectively) (Figure 4). Dysodensiol I (**42**) proved to be equally potent to the reference product methotrexate in inhibiting proliferation and inducing apoptosis in synoviocytes [19]. Dysodensiol F (**43**) is also an inhibitor of synoviocyte proliferation (IC_50_ = 11.8 µM), acting by binding to Toll-like receptor 4 (TLR4). This compound and related tricyclic guaiane sesquiterpenes can be obtained by total synthesis [77]. Recently, it has been used as a template for the synthesis of a library of about 100 derivatives bearing a double-ring conjugated enone scaffold. This effort led to the identification of potent compounds (IC_50_ = 2.6–2.8 µM) that show a good affinity for TLR4 and are orally active in an in vivo model of rheumatoid arthritis in rats. The most active compounds in the series were found to be equally active to methotrexate, markedly reducing the production of IL-6 and TNF-α in the serum, and devoid of apparent toxicity [78]. The series has been extended recently with the discovery and characterization of dysodensiols J, K, and L (**44**–**46**) from the stem of the plant, together with the related product aphanamol II (**47**). Dysodensiol K (**45**) is a robust inhibitor of synoviocyte proliferation, about four times more potent than aphanamol II (**47**) (IC_50_ = 6.3 and 26.6 µM, respectively) [79]. The dysodensiol compounds must contribute substantially to the anti-arthritis action of *F. oldhamii* extracts.

Other products isolated from the stems of *F. oldhamii* have been mentioned in publications in Chinese, such as asimilobine (**48**), laurotetanine (**49**), isocorydine (**50**), anolobine (**51**), N-methylbuxifoline (**52**), piperumbellactam A (**53**), goniopedaline (**54**), and salutaridine (**55**), but their specific contributions to the pharmacological activity of the plant extract have not been investigated (Figure 4) [20]. The promorphinan alkaloid salutaridine (**55**) (also known as sinoacutine) is a precursor to morphine in the opium poppy plant. The case of the isoquinoline alkaloid isocorydine (**50**) (also known as artabotrine and luteanin) is interesting because this product was recently shown to display marked anti-inflammatory properties and to protect mice from acute lung injury. Specifically, isocorydine (**50**) reduced the expression of pro-inflammatory IL-6 and attenuated the phosphorylation of p65 and JNK in bone-marrow-derived macrophages [80]. It represents a potent anti-inflammatory agent capable of regulating pro-inflammatory cytokine release via the inhibition of NFκB p65 translocation into the nucleus [81].

### 2.3. Natural Products from the Leaves

Various volatile sesquiterpenes have been characterized using an oil prepared from the leaves of *F. oldhamii*, principally γ-cadinene (**56**) (27.2%), β-caryophyllene (**57**) (23.7%), and β-ocimene (**58**) (10.2%) (Figure 4). The same type of terpenes can be found in the leaves of various Fissistigmatis species [21,82]. The dominance of sesquiterpenes was underlined in another study comparing the compositions of essential oils from the leaves of six Vietnamese species of *Fissistigma* [83].

### 2.4. Natural Products from the Fruits

Semi-volatile organic compounds can be found in different parts of the plants, such as the leaves but also the fruits. About 25 compounds were identified by capillary gas chromatography–mass spectrometry (GC-MS) using an oil prepared from fresh fruits of *F. oldhamii*, notably oleamide (**59**) ((Z)-9-octadecenamide) as a major constituent (21.7%), together with methyl-9-octadecenoate (7.6%), methyl hexadecanoate (6.7%), methyl-9-oxo nonanoic (6.6%), and phthalic acid (6.2%) [24]. The major product, oleamide (**59**), can be found in other seed essential oils; it is a strong antioxidant with marked superoxide-anion-scavenging capability [84]. Methyl 9-octadecenoate is a compound known for its insect deterrent activity and is most active against the settling of the aphids Myzus persicae and Rhopalosiphum padi (EC_50_ = 16 μg and 35 μg/cm^2^, respectively) [85]. Hexadecanoic acid and its methyl ester are also known as larvicidal products. These observations suggest that this fruit-based oil could be used in the control of pest aphids in agriculture, for example.

**Figure 5:**
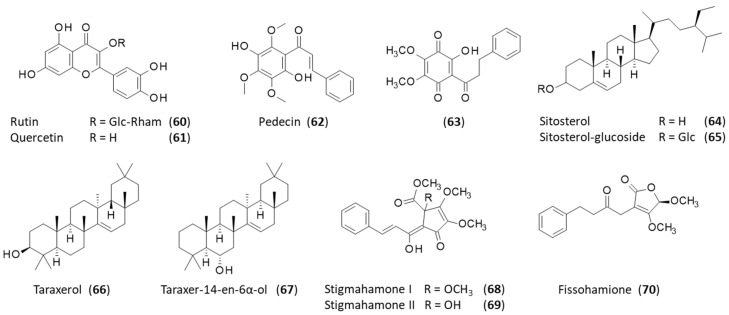
Structures of compounds **60**–**70**.

Flavonoids have been characterized from the fruits using a methanol extract. The products include classical flavanols such as rutin (**60**) and quercetin (**61**) (Figure 5), but also flavanones such as 6-hydroxy-5,7,8-trimethoxy flavanone and chalcones such as 2′,5′-dihydroxy-3′,4′,6′-trimethoxy chalcone [25]. This chalcone, better known as pedicin (**62**), is an antimitotic agent initially isolated from Didymocarpus pedicellata [23] and subsequently found in Fissistigma lanuginosum (Hook. f. & Th.) Merr. It was found to inhibit tubulin assembly into microtubules with an IC_50_ value of 300 µM, making it much less potent than the reference compound vinblastine (IC_50_ = 4 µM) [86]. Nevertheless, it is a rarely described compound worthy of further investigation. Another chalcone derivative has been recently described, namely, 4′,5′-dimethoxy-2′-hydroxy-3′,6′-quinodihydrochalcone (**63**), identified in the dried stems of *F. oldhamii* var. *longistipitatum*, and has revealed modest antiproliferative action against HepG2 hepatocytes (IC_50_ = 10.8 μM) [13].

Two main steroids were identified in the methanolic fruit extract: β-sitosterol (**64**) (cupreol) and a glucoside derivative (**65**) [25,86]. β-Sitosterol-3-O-β-D-glucopyranoside (**65**) is an inhibitor of mammalian DNA polymerase lambda (IC_50_ = 9.1 µM for intact Pol-λ) and can be found in diverse plants, such as in the brown skin of onions (*Allium cepa* L.). It inhibits the polymerase in a non-competitive manner (with respect to both DNA template–primer and dNTP substrates), probably by binding to the proline-rich N-terminal region of the protein [87]. It is also an inhibitor of sortase (IC_50_ = 18.3 µg/mL), an enzyme that is present on the cell surface of certain bacteria (including pathogenic Staphylococcus aureus) and that plays a key role in bacterial virulence. The inhibitory activity is supported by the glucopyranoside side chain because β-sitosterol is totally inactive against this enzyme [88]. Sortase A inhibitors are actively sought to combat superbug infections [89].

Two triterpenoids have also been identified in the fruits of *F. oldhamii*: taraxerol (**66**) and the derivative taraxer-14-en-6α-ol (**67**) [87]. Taraxerol (**66**) is an oleane-type pentacyclic triterpenoid (also known as alnulin, skimmiol, or tiliadin) with anti-inflammatory and cardioprotective activities [22]. It is a defense agent frequently found in higher plants and an orally active compound, potentially useful in treating cancer and other inflammatory diseases [90,91].

### 2.5. Natural Products from the Seeds

Two rare cyclopentenone derivatives, stigmahamones I (**68**) and II (**69**), have been isolated from the seeds of the plant upon extraction with methanol [16]. Recently, stigmahamone I (**68**) has also been isolated from the roots and the fruits of the plant [8]. To our knowledge, these two products have not been found in any other plants. They bear a structural similarity to the anti-neuroinflammatory agent linderone, a cyclo-pentenedione from Lindera erythrocarpa [92]. The related furanone derivative designated fissohamione (**70**) has also been found in *F. oldhamii* seed extract, but its bioactivity, if any, is unknown at present (Figure 5) [27].

## 3. Discussion

The plant *Fissistigma oldhamii* (Hemsl.) Merr. has long been used in traditional medicine for the treatment of various human diseases. In most cases, the dried, powdered roots and stems are used as decoctions to treat rheumatism and associated pain and fever. Both the roots and *rhizomes* of *F. oldhamii* are used as traditional folk medicines for hemostasis, rheumatoid arthritis, and other inflammatory diseases [10]. They are effective in dispelling wind and dampness and promoting blood circulation. In TCM, the plant is referred to as Gua fu mu (Chinese name) or Tie zuan or Xun gu feng (local names) [93]. In most cases, *F. oldhamii* is combined with other plants to make multiherbal preparations, prepared as either decoctions, patches, or tablets, as described in a number of Chinese patents (Table 2). The medicinal applications are varied, ranging from the treatment of sciatica to dysmenorrhea, cholangitis, migraine, arthritis, and other ailments and diseases. The common theme is pain and inflammation. The treatments rely essentially on the use of the roots, the stems, and/or the leaves of the plant, generally in combination with other plant roots.

The emphasis is on the natural products isolated from the plant and their pharmacological properties. But the active form of the product (for example, the ionic and isomeric forms of the chemicals), its formulation, the delivery process (as a drinkable solution or through inhalation or skin permeation, for example), and the best clinical practices (administration before/after a meal, etc.) are also important parameters to consider. They can affect the efficacy or safety of the product. In addition, the part of the plant used is often (but not always) indicated, but the exact preparation process is rarely given (use of fresh, dried, smashed, burned, or boiled materials; use of cold/hot water; in the presence of oil or fat; mixed with other plants; the duration of extraction/maceration/infusion; etc.). These are technical details not always mentioned in studies (publications and patents). The details of traditional recipes, which are nevertheless essential, are often transmitted orally or kept secret. The patent applications (Table 2) provide general information, but in some cases, it would be useful to have access to the precise recipe to better appreciate the exact nature of the ethnomedicine. Like synthetic products, plant-based medicinal products can exert negative side effects alongside the desired therapeutic effects. In some cases, the exact knowledge of the product formulation and the conditions of its use can facilitate the assessment of risks and benefits.

Over the past fifty years, the phytochemical content of the medicinal plant *F. oldhamii* has been analyzed to identify the active natural products at the origin of its anti-inflammatory, antioxidant, and immune-suppressive actions. The list of bioactive products has been continuously refined, from a dozen compounds in the 1980s to about 100 bioactive molecules inventoried today in all parts of the plant [20,94]. In 2021, Hu and coworkers used mass spectrometry to identify 54 compounds commonly present in the roots, stems, leaves, fruits, and insect galls, plus about 30 molecules present in one or more parts [8]. Recently, 64 compounds (44 alkaloids and 20 flavonoids) were identified in the related variant *F. oldhamii* var. *longistipitatum* [95]. Here, we have identified the most active compounds reported thus far. Many probably remain to be discovered in this medicinal plant. The main categories of active molecules are alkaloids, notably aporphine alkaloids, which are particularly abundant in Annonaceae in general [96].

**Figure 6:**
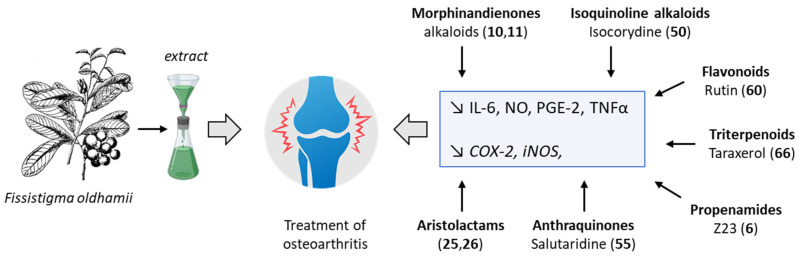
Decoctions from *Fissistigma oldhamii* are used to treat osteoarthritis and rheumatoid arthritis. Multiple anti-inflammatory natural products found in plant extracts may be at the origin of the activity through the regulation of different mediators of inflammation, such as cytokines (IL-6, TNF-α) or genes implicated in the inflammatory process (iNOS, COX-2).

The anti-inflammatory action of *F. oldhamii* extracts likely results from the unique combination of natural products present in the plant roots and stems, rather than from a specific major molecular entity. Several anti-inflammatory products have been identified, including morphinandienone alkaloids, aristolactam derivatives, triterpenoids, and others with various actions, as schematized in Figure 6. The combination of flavonoids, anthraquinones, and aporphine alkaloids can lead to robust anti-inflammatory effects. For example, the combination of the anthraquinone emodin or sinoacutine with the flavonoid acacetin has been recently shown to exert potent anti-inflammatory effects that are superior to those of the individual components [97]. Comparable effects may occur when using an extract of *F. oldhamii*, which also contains similar flavonoids and anthraquinones, notably sinoacutine (salutaridine, **55**) [14,20]. The propenamide derivative Z23 is also an important contributor to the anti-inflammatory effect through its capacity to reduce the expression of the cytokines IL-6, TNF-α, and IFN-γ, in addition to downregulating genes like COX-2 and iNOS [12,39]. The flavonoids, phenolics, saponins, and alkaloids from *F. oldhamii* participate in the resolution of inflammation, as observed with other TCM preparations [98]. But more work is needed to abridge the ethnobotany and phytochemistry of *F. oldhamii* in order to determine the origin of the anti-inflammatory activity of this Annonaceae species.

The medicinal properties of *F. oldhamii* have been known for a long time, but this useful plant has been much less investigated than other *Fissistigma* species and other plants of the Annonaceae family. The pharmacological characterization of the traditional medicines derived from this plant undoubtedly deserves further research to better exploit the derived products and their therapeutic effects.

## Figures and Tables

**Table 1:** Natural products isolated from the different parts of *Fissistigma oldhamii* (Hemsl.) Merr.

Plant Parts	Main Natural Products	References
Roots	Aristolactams A-B; corytuberine; physcion; stigmastanone; Z23	[10,11,12]
Stems	Aristololactams AII-AIIIa, BII, FI-FII, GI-GII; calycinine; duguevalline; dysodensiols G-L; enterocarpam; fissistigamides A-B; fissistigine A; fissistigmine; fissistyramine; fissoldine; goniothalactam; isocorydine; isopedicin; lyciumide A; noraristolodione; norcepharadione B; oldhamactam; O-methylmoschatoline; oxocalycinine; oxodiscoguattine; oxoxylopine; piperolactams A-C; romucosine; stigmalactam; velutinam; xylopine	[10,13,14,15,16,17,18,19,20]
Leaves	γ-Cadinene; β-caryophyllene; β-ocimene	[21,22]
Fruits	Methyl-octadecenoate, methyl hexadecanoate, methyl oxononanoic acid; octadecenamide; pedicine; quercetin; rutin; β-sitosterol; taraxerol; taraxerenol	[23,24,25,26]
Seeds	Fissohamione; stigmahamones I–II	[8,16,27]

**Table 2:** Chinese patents including *Fissistigma oldhamii* and their applications.

Patent (CIB #)	Title	Application
**A23L 1/29** (201510503771.7) CN-24.05.2017	*Fissistigma oldhamii* wind-dispelling dampness-eliminating soup base and preparation method.	Method to prepare a plant soup containing *F. oldhamii*, useful in promoting blood circulation, arresting pain, and relaxing tendons
**A61K 36/896** (202111278310.6) CN-14.12.2021	Traditional Chinese medicine patch for treating sciatica and preparation method of traditional Chinese medicine patch	A recipe to prepare a TCM patch for the treatment of sciatica, including roots of *F. oldhamii* and many other plants.
**A23F 3/14** (201510482935.2) CN-24.05.2017	*Allophylus viridis* Radlk wind-dispelling dampness-eliminating tea and preparation method.	Method to prepare a dampness-eliminating tea including roots of *F. oldhamii* and many other plants. The preparation is used to promote blood circulation to remove blood stasis and relax tendons.
**A61K 36/899** (201610201188.5) CN-22.06.2016	Traditional Chinese medicine composition for treating qi-blood weakness type dysmenorrhea.	Composition and preparation of a TCM tablet used to treat qi-blood weakness. It includes *F. oldhamii* and many other plants, used together to treat dysmenorrhea.
**A61K 36/898** (201410322104.4) CN-17.09.2014	Chinese herba preparation capable of treating qi and blood deficiency osteoarthritis and preparation method.	Method to prepare an herbal mixture of *F. oldhamii* and other plants, used to reduce phlegm and for dissipating stasis, dispelling dampness, and dredging collaterals.
**A61K 36/87** (102016000646482) CN-23.11.2016	Medicine for treating hepatitis containing herba senecionis scandentis.	A multiherbal TCM containing *F. oldhamii*, used internally and externally to combat hepatitis (prevention and treatment).
**A61K 36/898** (201710376789.4) CN-15.09.2017	Traditional Chinese medicine patch for treating hyperostosis.	*F. oldhamii* is included in a patch developed to promote nourishing yin, tonify the kidneys, expel wind, remove cold, remove dampness, and relieve pain.
**A61K 36/899** (201310663260.2) CN-05.03.2014	Traditional Chinese medicine for treating acute suppurative cholangitis.	Recipe for a multiherbal preparation including *F. oldhamii*, designed to treat suppurative cholangitis.
**A23L 1/39** (102015000316564) CN-04.01.2017	*Schefflera arboricola* wind dispelling and pain stopping oyster seafood soup materials and preparation method.	Recipe for a tasty oyster seafood soup including *F. oldhamii*, used for dispelling wind and stopping pain.
**A61K 36/9066** (201410548829.5) CN-07.01.2015	Traditional Chinese medicine preparation for treating apoplexy sequela due to vital energy deficiency and blood stasis and preparation method of traditional Chinese medicine preparation.	A TCM preparation containing *F. oldhamii* with various effects: benefiting vital energy, activating blood, strengthening healthy energy, and eliminating evil. For the treatment of the apoplexy sequela due to vital energy deficiency and blood stasis.
**A61K 36/8945** (201510138462.4) CN-15.07.2015	Qi and blood deficiency type migraine treating drug and preparation method.	A TCM preparation containing *F. oldhamii* for the treatment of qi- and blood-deficiency-type migraine.
**A61K 31/575** (102016000273430) CN-31.08.2016	Application of steroids in preparing drugs for treating rheumatoid arthritis.	Method to extract steroids from *F. oldhamii* and their use in treating rheumatoid arthritis.
**A61K 36/84** (201710801775.2) CN-15.12.2017	Decoction medicine for treating cervical and lumbar spine disease and preparation method.	Preparation method for a multiherbal TCM decoction including *F. oldhamii* used to treat cervical and lumbar spine diseases.
**A61K 36/899** (201710436824.7) CN-22.09.2017	Facial paralysis treating traditional Chinese medicine composition.	A TCM preparation containing *F. oldhamii* for treatment of facial paralysis with the following effects: wind evil dispelling, collateral dredging, heat clearing, blood circulation activation, phlegm dissipating, and nutrient qi regulation.

Major patents identified using PatentScope “https://patentscope.wipo.int” (accessed on 6 September 2023) using the search term *Fissistigma oldhamii*.

## Data Availability

Data are contained within the article.
